# A Multidisciplinary Structural Approach to the Identification of the *Haemophilus influenzae* Type b Capsular Polysaccharide Protective Epitope

**DOI:** 10.1021/acscentsci.3c01515

**Published:** 2024-02-22

**Authors:** Francesca Nonne, Lucia Dello Iacono, Sara Bertuzzi, Luca Unione, Daniela Proietti, Nathalie Norais, Immaculada Margarit, Roberto Adamo, Jesús Jiménez-Barbero, Filippo Carboni, Maria Rosaria Romano

**Affiliations:** † GSK Vaccines Institute for Global Health, 53100 Siena, Italy; ‡ GSK, 53100 Siena, Italy; § CIC bioGUNE, Basque Research Technology Alliance, BRTA, Bizkaia Technology Park, 48160 Derio, Spain; ∥ IKERBASQUE, Basque Foundation for Science and Technology, Euskadi Plaza 5, 48009 Bilbao, Spain; ⊥ Department of Organic & Inorganic Chemistry, Faculty of Science and Technology, University of the Basque Country, 48940 Leioa, Spain; # Centro de Investigación Biomédica En Red de Enfermedades Respiratorias, 28029 Madrid, Spain

## Abstract

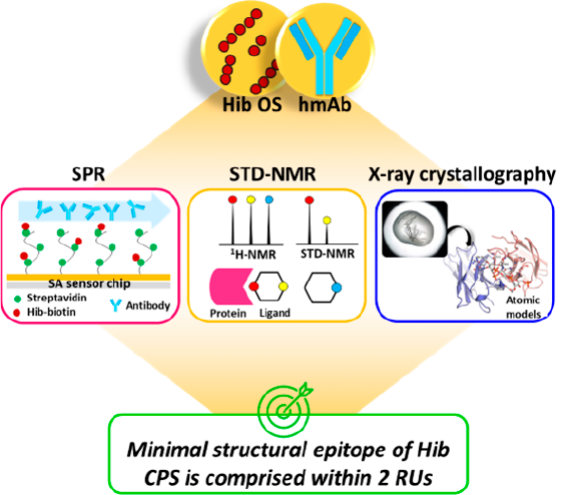

Glycoconjugate vaccines so far licensed are generally composed of a native or size-reduced capsular polysaccharide conjugated to carrier proteins. Detailed information on the structural requirements necessary for CPS recognition is becoming the key to accelerating the development of next-generation improved glycoconjugate vaccines. Structural glycobiology studies using oligosaccharides (OS) complexed with functional monoclonal antibodies represent a powerful tool for gaining information on CPS immunological determinants at the atomic level. Herein, the minimal structural epitope of *Haemophilus influenzae* type b (Hib) CPS recognized by a functional human monoclonal antibody (hmAb) is reported. Short and well-defined Hib oligosaccharides originating from the depolymerization of the native CPS have been used to elucidate saccharide–mAb interactions by using a multidisciplinary approach combining surface plasmon resonance (SPR), saturation transfer difference-nanomagnetic resonance (STD-NMR), and X-ray crystallography. Our study demonstrates that the minimal structural epitope of Hib is comprised within two repeating units (RUs) where ribose and ribitol are directly engaged in the hmAb interaction, and the binding pocket fully accommodates two RUs without any additional involvement of a third one. Understanding saccharide antigen structural characteristics can provide the basis for the design of innovative glycoconjugate vaccines based on alternative technologies, such as synthetic or enzymatic approaches.

## Abstract 2

Short, well-defined Hib oligosaccharides originating from depolymerization of the native capsular polysaccharide were complexed with a human functional monoclonal antibody to gain information on the minimal structural epitope of Hib CPS at the atomic level.

## Introduction

*Haemophilus influenzae* is a Gram-negative coccobacillus that colonizes the nasopharynx in healthy adults, children, and infants.^1^ It can be found in unencapsulated (NTHi) or encapsulated forms classified into six serotypes (*a*–*f*) according to the capsular polysaccharide (CPS) chemical structure. *Haemophilus influenzae* serotype b (Hib) is the predominant form that infects mostly children and immunocompromised individuals. Hib causes severe invasive infections such as epiglottitis, sepsis, pneumonia, and meningitis.^2^ CPS of Hib consists of polyribosyl-ribitol-phosphate (PRP) repeating units and represents its most important virulent factor.^3^ As for the majority of polysaccharide-based vaccines, Hib purified PRP is immunogenic in adults but fails to induce protective antibodies in infants.^4,5^ In 1987, Hib PRP was conjugated to diphtheria toxoid (DT) protein, which improved its immunogenicity, particularly in young children, resulting in the first glycoconjugate vaccine ever developed. Currently, Hib glycoconjugate vaccines with different carrier proteins are now available on the global market both in monovalent formulations and in combination with other antigens.^1^ Furthermore, Hib has been the target of the first synthetic glycoconjugate vaccine, Quimi-Hib, composed of a synthetic antigen made by an average of seven repeating units of PRP conjugated to tetanus toxoid (TT).^6^ This finding drew attention to the concept that glycoconjugate oligosaccharides long enough to cover the native polysaccharide epitope could elicit comparable or even better immune responses than a native polysaccharide conjugate product.^7−9^ The use of oligosaccharides provides a chemically defined composition of the vaccine which is advantageous to the manufacturing process.^10^

Over the years, Hib oligosaccharides obtained both from the depolymerization of natural polysaccharides^7,8,11,12^ and by chemical synthesis^13−15^ have been used in studies aimed at identifying the minimal epitope able to induce an immune response. A clinical study in 1987 demonstrated that a DT-conjugate formed by an oligosaccharide with an average length of 20 RU was immunogenic in 1-year-old infants.^16^ In 1997, Chong et al. reported a comparison between TT-conjugates of synthetic Hib dimer and trimer oligosaccharides tested in a rabbit model. The study clearly indicated that at least three repeating units were required to achieve immunogenicity.^15^ Conversely, Pillai et al. observed, through competitive ELISA analysis, that two repeating units were able to inhibit antibody binding to the native polysaccharide, suggesting that even DP2, although containing only four monosaccharides, might present an optimal length for the complete filling of the antibody binding sites, probably due to the presence of two phosphate groups as two extra residues for a total of six.^9^ More recently, Baek and co-workers compared *in vivo* synthetic oligomers ranging in length from a tetramer up to a decamer conjugated to a CRM_197_ protein. Their results indicated that the tetramer resembled the longer polysaccharide in terms of immunogenicity and antibody recognition.^3^ Nevertheless, in addition to saccharide length, other variables such as the saccharide:protein ratio may play an important role in the immunogenicity of glycoconjugate vaccines.^17^ Anderson et al. reported that Hib oligosaccharides conjugated to carrier proteins with an optimal degree of glycosylation may be more likely to activate T-helper cells than conjugates obtained from the corresponding polysaccharide.^11^ A high degree of glycosylation allows for multiple displays of the epitopes, thus compensating for the shorter saccharide length through the so-called multivalence effect. This effect suggests that short oligosaccharide fragments, when exposed many times on the protein surface, can lead to optimal antibody responses.^18,19^ A comparison between Hib oligosaccharide conjugates of different lengths demonstrated that an average degree of polymerization of 7 (avDP7) with a high number of saccharide chains loaded onto the carrier protein was more active in infants than longer oligosaccharide conjugates with a lower loading.^7^

Elucidating the polysaccharide minimal epitopes recognized by functional antibodies mediating protection from infection is crucial to guiding the design of optimized carbohydrate-based vaccines. In the past few years, structural studies aimed at mapping polysaccharide antigen determinants and epitope conformations have been applied to different bacteria. The present study has been designed to apply a multidisciplinary approach by combining SPR, STD-NMR, and X-ray crystallography methodologies to unravel the structural antigenic determinants of Hib CPS.

## Results

### Selection of Hib Oligosaccharides for Structural Studies

To characterize the interaction between Hib CPS and functional antibodies at the atomic level, a recombinant monoclonal antibody and its corresponding fragment antigen-binding (Fab) region were produced based on the published nucleotide sequence of a functional human IgG2 mAb specific to Hib CPS, named CA4.^20^ CA4 belongs to one of the most abundant families of anti-Hib antibodies derived from the VκIII (A27) gene^21^ and was selected on the basis of its *in vitro* and *in vivo* functional activity against Hib bacteria.^20^

**Figure 1:**
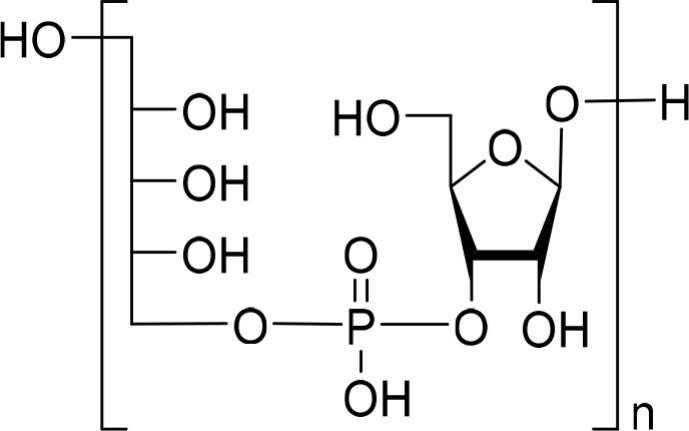
Hib oligosaccharide structure obtained by the hydrolysis of native CPS.

In parallel, a set of oligosaccharide fragments were generated through acid hydrolysis of the natural Hib polysaccharide as already described^22^ and purified by ion exchange chromatography. The acidic treatment cleaved the glycosidic linkage between ribose and ribitol units^22,23^ (Figure 1), as confirmed by NMR analysis showing that hydrolysis generated ribose as a reducing sugar. This finding was confirmed by the absence of a terminal phosphomonoester form in the ^31^P NMR spectra (Figure S1A). Oligosaccharides composed of 2 to 5 repeating units (DP2-DP5), as confirmed by electrospray ionization-mass spectrometry (ESI-MS) analysis (Figure S1B), were selected for structural studies.

First, competitive SPR experiments were performed to interrogate the inhibitory capacity of the different oligosaccharide fragments against CA4 hmAb binding to a Hib polysaccharide. Specifically, Hib DP2, DP3, DP4, and DP5 oligosaccharides were used as competitors of the binding between the hmAb and a biotinylated Hib polysaccharide (avDP80) immobilized on a streptavidin (SA) chip. The final percentage of hmAb binding inhibition was plotted against the concentration of each of the tested oligosaccharides.

**Figure 2:**
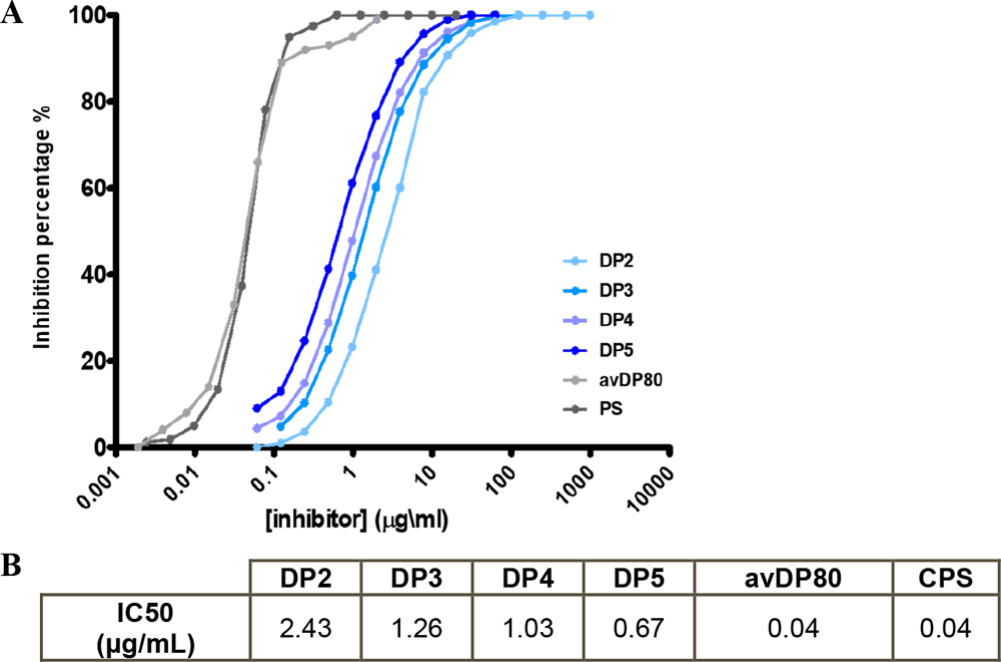
A) Competitive SPR curves showing the specific length-dependent recognition of DP2-DP5 oligosaccharides by the CA4 hmAb. Hib avDP80 polysaccharide and Hib native polysaccharide (PS) were used as positive controls in the competition. B) Measured IC50 values for the interaction of CA4 hmAb with Hib oligosaccharides and polysaccharides.

SPR data showed that all tested oligosaccharides competed against the CPS for CA4 hmAb binding, indicating that DP2 already contained the minimal Hib CPS portion necessary to completely inhibit the antibody-CPS recognition. Moreover, the corresponding binding affinities reflected the length of the saccharide chains with derived IC50 values systematically lower for larger OSs (Figure 2). The length-dependent affinity of the different fragments could derive from (i) bivalent mAb interaction, which results in an avidity effect,^24^ (ii) statistical rebinding, or (iii) a better presentation of the surface-extended OSs. To address this point, we used a different assay in which *K*_D_ affinities for the Fab fragment were evaluated for biotylinated Hib DP2, DP3, DP4, and avDP80 (Figure S2A, Table 1). In the former competitive SPR experiments, the biotinylated CPS was immobilized on an SA chip while the intact oligosaccharides complexed with the hmAb were injected over the chip in a continuous flow. Instead, for the kinetic experiments, each oligosaccharide was biotinylated to achieve immobilization on a SA chip. The biotinylation reaction consists of the covalent binding of biotin to the terminal reducing end of the oligosaccharides, which obviously impacts the length of each fragment, as the ribose of the terminal RU is locked in its open form (Figure S2B), with the consequent absence of ribose at the reducing end. The biotinylated fragments consisted of intact RUs terminating in ribitol phosphate linked to the open chain ribose-biotin. Consequently, biotinylated DP3 and DP4 contained 2 and 3 complete RUs and 1 terminal derivatized RU, respectively, while biotinylated DP2 contained only 1 complete RU and 1 derivatized RU. For DP2, it was not possible to carry out a kinetic experiment with the Fab, as the derivatized fragment was probably too short to cover the epitope, confirming that at least two consecutive complete repeating units were needed for specific mAb recognition.

**Table 1:** *K*_D_ Mean Affinities for Binding to Fab CA4 Calculated over Three Experiments for Each OS

Hib oligosaccharides	*K*_D_ mean (M)
DP3-Biotin	5.01 × 10^–6^
DP4-Biotin	8.01 × 10^–7^
avDP80-Biotin	4.00 × 10^–7^

Biotinylated Hib DP3 and DP4 showed high Fab affinities with *K*_D_ values not very dissimilar from that of the avDP80 polysaccharide. These results suggested that two and three repeating units represented optimal lengths for structural studies aimed at dissecting the interaction with the selected hmAb at the atomic level.

### Characterization of CA4 hmAb–Hib DP2 and DP3 Interaction by STD-NMR

**Figure 3:**
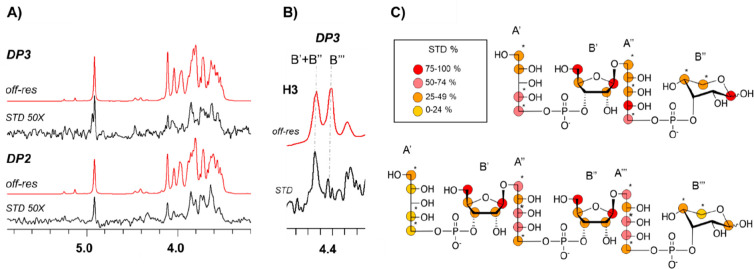
A) ^1^H-STD-NMR experiments were performed for the complexes of Hib DP2 and DP3 with the CA4 hmAb. Off-resonance spectra (in red) and corresponding STD-NMR spectra (in black). B) Expansion of the STD-NMR spectrum (in black) and of the corresponding off-resonance (red) relative to the H3 signals of the ribose units of DP3. C) Representation of the epitope map disclosed by the analysis of the STD-NMR data of Hib DP2-CA4 and Hib DP3-CA4 hmAb complexes. A color legend associated with the STD% values is reported. Asterisks are used to indicate overlapping and partially overlapping NMR signals for which only a rough estimation of the relative STD contribution was possible. For convenience, ribitol and ribose moieties of the OS fragments are individually indicated as A′, B′, A′′, B′′, A′′′, and B′′′ starting from the ribitol of the first repeating unit.

Saturation transfer difference NMR (^1^H STD-NMR) experiments were performed to deduce the key structural features of the binding of Hib DP2 and Hib DP3 oligosaccharides in solution to the CA4 hmAb at atomic resolution.^25−27^ The resulting STD-NMR spectra showed the binding of both DP2 and DP3 OSs. The relative STD-NMR signal intensities were used to define the corresponding binding epitopes (Figure 3).

In solution, the reducing terminal d-ribose moiety of both DP2 and DP3 exists as a complex equilibrium mixture of α/β pyranose and furanose forms, which rapidly interconvert through the mutarotation process. The corresponding ^1^H–^13^C HSQC anomeric signals were integrated, and the contribution of each of the two forms was then determined. The predominant form was Rib*p*β (58%), followed by Rib*p*α (26%), Rib*f*β (15%), and Rib*f*α (7%), in agreement with previously reported data in solution^28,29^ (Figures S3 and S4). Given the higher population of the Rib*p*β in solution, the STD-NMR analysis refers to DP2 and DP3 OSs having a β-pyranose ring as the reducing end. Consistently, the alternative forms, Rib*p*α, Rib*f*β, and Rib*f*α, poorly contribute to the STD spectrum (Figures 3, S5, S6, and S7).

For DP2, the strongest STD-NMR signals arose from the central part of the molecule (Figures 3 and S5). In particular, the H1, H5, and H5′ protons of internal ribose, together with the H4, H5, and H5′ protons of the internal ribitol, displayed similar STD effects that ranged between 75 and 100% of the maximum STD relative intensity. In contrast, the STD signals of the terminal ribitol moiety were rather weaker. In particular, the corresponding H1, H2, H4, and H5 protons showed relative STD intensities that ranged between 25 and 74%. Medium-weak STD intensities were detected for H4 and H5 of the Rib*p*β at the reducing end.

Overall, these data clearly indicated that the binding epitope of DP2 was mainly defined by the central ribose and ribitol units, while the terminal ribitol (at the nonreducing end) and the Rib*p*β (at the reducing end) were more exposed to the solvent.

Next, the epitope of the DP3 oligosaccharide was determined (Figures 3 and S6). The resulting STD-NMR spectrum was very similar to that obtained for DP2, indicating a very similar binding mode. Nonetheless, noteworthy differences existed. In DP3, the H3 protons of the ribose sugars appeared in a spectral region devoid of other signals, which allowed for clear discrimination of their relative STD contributions (Figure 3B). The NMR signals corresponding to the two internal Rib*f* moieties showed clear STD intensities. On the contrary, the H3 of the ribose at the reducing end did not provide any STD contribution. These data indicated that in the longer hexasaccharide (DP3) the two internal ribose units were in close contact with the hmAb while the reducing end was solvent-exposed. Fittingly, the relative intensity of the STD from H4 and H5 of the different ribose units further supported this conclusion (Figure S6).

Taken together, this analysis indicates that the internal ribose and ribitol chains directly engage the CA4 hmAb binding site while the terminal units extend outside the binding pocket (Figure 3).

### Three-Dimensional Structures of Human Fab CA4 Complexed with DP2 and DP3 Oligosaccharide Fragments

To gain further insights into the molecular basis of recognition, Hib OS fragments were crystallized and complexed with the Fab fragment derived from CA4 monoclonal antibody. Crystals of DP2 and DP3 oligosaccharides complexed with the human Fab CA4 were determined in the orthorhombic space group *C*222_1_ at 2.29 and 2.74 Å resolution, respectively, with a single Fab copy in the asymmetric unit. The structures were refined to final *R*_*work*_/*R*_*free*_ values of 22.6/27.0% (Fab-DP2 complex) and 22.6/27.7% (Fab-DP3 complex).

**Figure 4:**
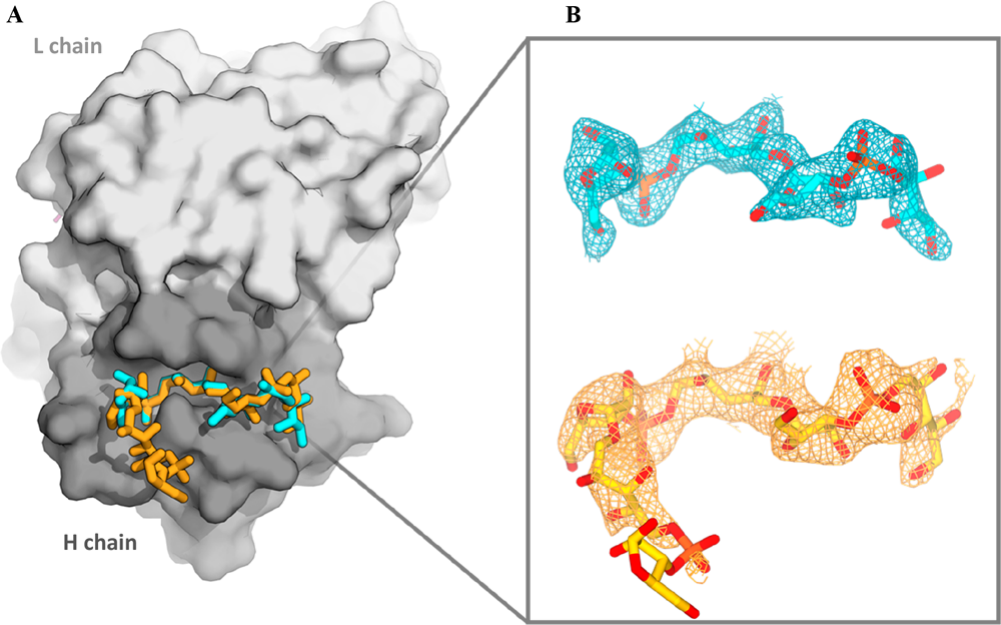
A) Superposition of the Fab CA4/DP2 and Fab CA4/DP3 X-ray structures. The Fab is shown as a surface with a gray H chain and a white L chain, and the two OS fragments are shown as sticks (DP2-cyan and DP3-yellow). B) Final (2Fo – Fc) electron density maps of DP2 and DP3 OS were contoured at 1.5σ. OS structures are represented as sticks colored by elements (C atom in cyan/yellow, P atom in orange, and O atom in red).

Superposition of the two complexes revealed that the overall fold of the antibody was essentially identical, irrespective of the length of the bound OS, exhibiting a root-mean-square deviation (RMSD) of 0.32 Å for the pairwise superposition of 435 Cα atoms (Figure S8, where ribitol (Rib-ol) and ribose (Ribf) moieties of the OS fragments are individually indicated as A′, B′, A′′, B′′, A′′′, and B′′′ starting from the ribitol of the first repeating unit). Fab CA4 heavy (H) and light (L) polypeptide chains showed clear electron density in both X-ray structures. As concerns the OS fragments, unambiguous electron density was observed for the entire DP2 length. For the DP3 OS, after the first cycles of refinement, clear electron density was visible for units A′B′-A′′B′′ and on the Rib-ol A′′′. No density appeared on the floppy Ribf B′′′ or the phosphodiester bridging Rib-ol A′′′-Ribf B′′′, likely protruding outside the antibody cleft occupied by the DP2 (Figure 4). For completeness, both Rib-ol A′′′-Ribf B′′′ were included in the final model with zero site occupancy and not refined.

**Figure 5:**
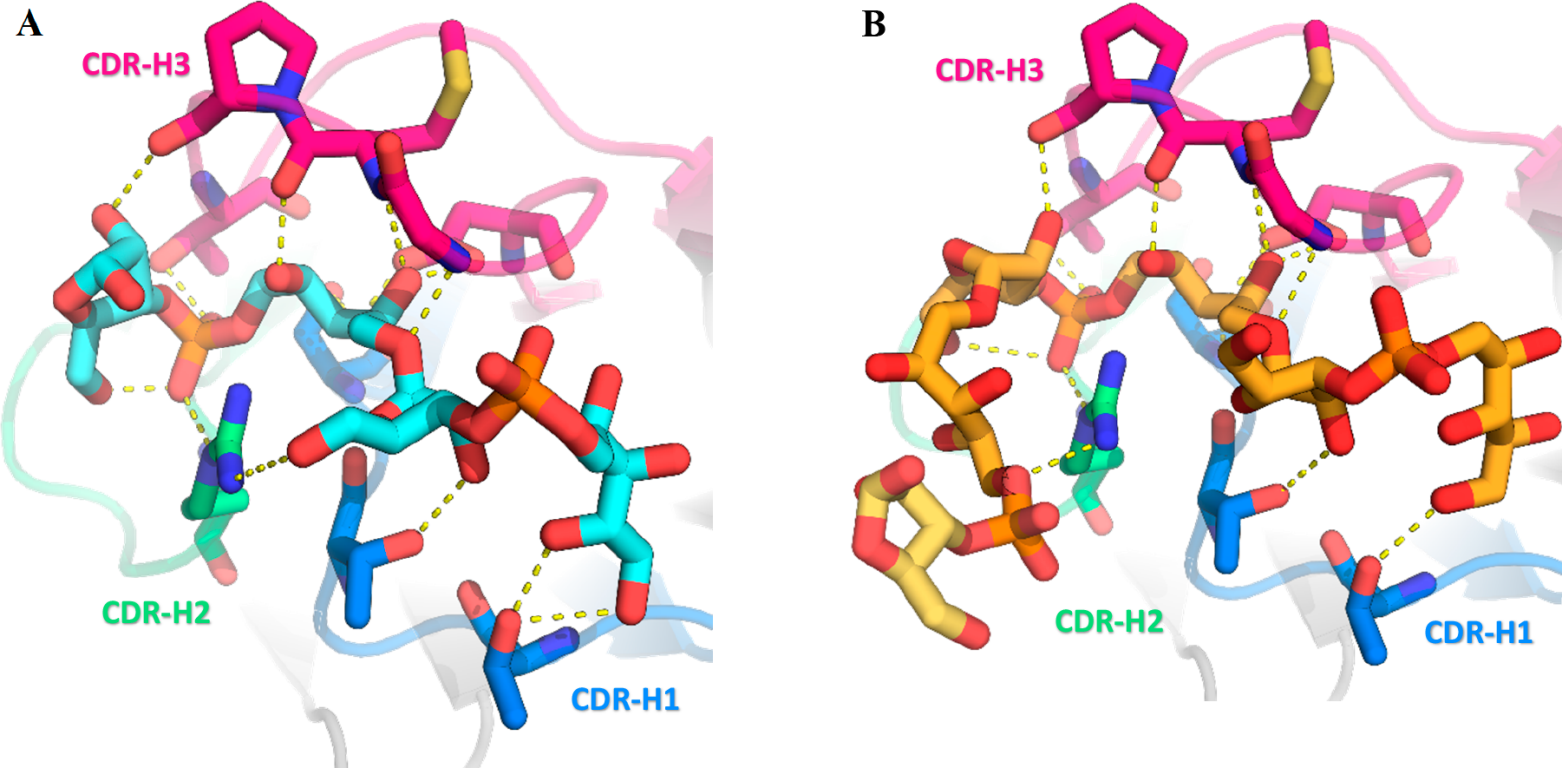
Zoomed-in view of the most relevant interactions established by A) DP2 (cyan C sticks) and B) DP3 (yellow C sticks) with H-chain CDR residues. CDR residues are depicted as sticks, with a color-coding scheme reflecting the CDR classification (CDR H1, blue; CDR H2, green; and CDR H3, hot pink).

In terms of antibody-saccharide recognition, the total surface area occluded from the bulk solvent upon the formation of the Fab-DP2 complex is ∼870 Å^2^ (360 Å^2^ from the Fab paratope and 510 Å^2^ from the glycan). DP2 is accommodated in a Fab small groove-shaped binding site delineated exclusively by the H-chain complementarity determining regions (CDRs) H1, H2, and H3. The long 16-residue CDR H3 (according to North annotation) is likely oriented to shield the epitope from interactions with the Fab L chain (Figure 5A). The DP2-Fab binding affinity is the result of contributions mainly arising from polar and electrostatic interactions, involving all major saccharide functional groups.

In further detail, Rib-ol A′ establishes two direct H-bonds with a Thr28 backbone and a side chain (CDR H1) through its hydroxyl groups at positions 1 and 2 while its OH groups at positions 3 and 4 are water-mediated bridged to Arg98 (CDR H3). The phosphodiester group between A′ and B′ is not involved in relevant interactions with the Fab, while the Ribf B′ interacts with Gly102, Thr31, and Arg53 through its OH-1, OH-2, and OH-5 groups, respectively. Moving to the most engulfed RU A′′B′′, the Rib-ol A′′ is stabilized by six direct HBs with CDR H2 and H3: the OH-2 group is within the HB distance from the Asp99 side chain and Met103 backbone, the OH-3 interacts with both the Ser33 backbone and Asp99 side chain, and the OH-4 group makes an HB with a Met103 backbone carbonyl and an Arg53 side chain. H-bonds of the central phosphodiester group A′′-B′′ are established with side chains of CDR residues Arg53 and Thr106. The Ribf B′′ moiety interacts with the Pro104 backbone of CDR H3 through its OH-2 while its OH-5 group is water-mediated bridged to Ser52 and Ser54 side chains. Noteworthy, the DP2 conformation is also stabilized by four intramolecular H-bonds, two of which involve the phosphodiester groups. The A′′-B′′ phosphodiester is bridged to the Ribf B′′ OH-5 group, while the A′-B′ phosphodiester approaches the Rib-ol A′ OH-4 group. Additionally, intramolecular interactions are also possible between the Ribf B′ OH-2 group and Rib-ol A′ OH-2.

In addition to H-bonds, Fab-DP2 binding is mediated by electrostatic interactions between the positively charged Fab binding pocket and the negatively charged phosphodiester moieties of the saccharide (Figure S9). Although different in length, the DP2 and DP3 oligosaccharides adopt very similar conformations when they are bound to the antibody. However, the Rib-ol A′ group shows slightly different orientations in the two structures, likely as a consequence of its flexibility. In support of this, B-factor values reveal that this moiety remains rather mobile, especially in DP2 (Figure S9).

As a consequence of the similar binding mode of the two OS fragments, most of the interactions established by DP2 are nearly identical in complex Fab-DP3 (Figures 5 and S10), strongly suggesting that the first two RUs represent the crucial anchoring core for Hib carbohydrate recognition. This consideration is completely in line with the STD-NMR results obtained, which showed the existence of almost identical binding modes for both DP2 and DP3. Notably, DP3 Rib-ol A′ engages the same interactions established by the same group in DP2, being able to contact both Thr28 and Arg98 residues as well as adjacent Ribf B′ still being able to bind Thr31. Regarding the second RU, the interactions of both Rib-ol A′′ and Rib-f B′′ are largely preserved. The only relevant difference between DP2 and DP3 takes place in Ribf B′, which loses its interaction with the Arg53 residue. As concerns the third RU of DP3, it extends out of the binding pocket, resulting in only one weak interaction with the antibody, i.e., a H-bond between the Rib-ol A′′′ OH-3 group and the Arg53 (CDR H2) side chain. This finding demonstrates that the third RU (especially the Ribf B′′′ group) is relatively free and mobile since it is not critical for antibody binding, explaining the diffuse electron density for this portion of the DP3 OS and the STD-NMR results.

## Discussion

Conjugate vaccines have been one of the major developments of the last 40 years.^30^ Many aspects can influence the immunogenicity of glycoconjugates, such as the saccharide:protein ratio, the conjugation strategy, the nature of the spacer and protein carrier, and the size of the saccharide moiety. Hence the identification of the bacterial polysaccharide minimal epitope is crucial to guiding the rational design of modern and efficacious glycoconjugates vaccines. Traditional glycoconjugates are composed of long poly- or oligosaccharides containing many copies of the repeating unit. However, immunogenic CPS epitopes involved in the interaction with specific antibodies usually comprise precise glycan structures, often not longer than six or eight sugar units (a 45-year-old paradigm established by Kabat),^31^ and in the literature, even oligosaccharides as short as di- or tetra-saccharides have been shown to possess the minimal structural requirements for raising functional antibodies.^32−34^ The development of advanced technologies applicable to the glyco field has made it possible to characterize the protective epitope of CPS at the atomic level, highlighting the fundamental structural characteristics in the interaction with functional antibodies. Recently, it has been demonstrated that five to six RUs contain the minimal structural and immunogenic epitope of *N. meningitidis* serogroup X (MenX) capsular polysaccharide.^35^ The study at the atomic level of the minimal epitope of group B *Streptococcus* type III revealed a sialylated epitope spread over two adjacent repeating units.^36^ The same methodology applied to *N. meningitidis* serogroup A identified the O-acetylated trisaccharide as the minimal antigenic epitope.^37^ In this study, we determined the molecular structure of a protective epitope of Hib CPS using a multidisciplinary approach including SPR, STD-NMR, and X-ray crystallography to obtain information on its structural antigenic determinants. For this work, the availability of short and well-defined oligosaccharides (DP2-DP5) was essential to characterizing the minimal saccharide epitope directly involved in binding of a protective and functional human monoclonal antibody, named CA4,^20^ as representative of the anti-Hib human functional Ab repertoire. SPR studies led to the elucidation of the antigenic minimal epitope, revealing that all Hib oligosaccharides used, including the shortest composed of only two repeating units, were well recognized by the protective hmAb with slight length-dependent behavior from DP2 to DP5 up to polysaccharide avDP80 and the native capsular polysaccharide. These findings guided STD-NMR studies on the DP2-hmAb complex, which confirmed the recognition of DP2 by the selected antibody and provided information on the protons of the sugar molecules more involved in the interaction in terms of proximity to the protein surface STD-NMR results, which confirmed the recognition of the DP2 antigen by the selected antibody, with an overall contribution of all of the sugar protons. In particular, the central ribitol and ribose appeared to be the closest portions to the binding pocket. Obtaining the crystal structure of the complex between oligos and the Fab fragment, not obvious in the presence of very flexible structures such as sugars, was fundamental to obtaining high-resolution information about binding interactions. Indeed, despite the power of the technique, relatively few crystal structures of carbohydrate-antibody complexes have been solved so far, with the majority of them describing interactions with murine or rabbit antibodies.^36−42^ To the best of our knowledge, only five X-ray complexes including human antibodies have been reported to date.^43^ None of these involved anti-Hib antibodies. Here we employed a Fab fragment derived from the full-length human CA4 IgG, belonging to the non-A2 family of anti-Hib mAbs, for cocrystallization studies with Hib oligosaccharide DP2 or DP3. The repertoire of human antibodies to Hib PS was extensively characterized and can be divided into A2, the most abundant family, and non-A2 antibodies based on the Vkappa gene. While the role of the L chain in antigen recognition for A2 Abs was known, where specific residues appear to be important for antigen binding,^21^ no information was available about non-A2 mAb.^20,21^ Comparison of the affinity-matured CA4 antibody with its corresponding germline precursors revealed that mutations were acquired by CA4 H and L chains,^20^ suggesting a possible involvement of both chains in antigen recognition. Surprisingly, the crystal structure of the Fab CA4-DP2 complex clearly showed that the antibody participates in a groove-type binding of the DP2 ligand using the three H-chain CDRs and without any involvement of its L chain. On the basis of our data, it is puzzling to explain the H-chain dominance shown by CA4, which is rather unusual for antiglycan antibodies. Further data are necessary to assess whether this is a peculiar feature shown by mAb CA4 or shown by other anti-Hib mAbs along with the functional implications of such an unusual recognition profile. Indeed, previous studies have already revealed that, despite the relative simplicity of glycan antigens compared to protein antigens, different anticarbohydrate antibodies can target distinct epitopes.^44,45^ As an example, antibodies against chlamydial LPS antigen showed various epitopes, and this has been correlated with the evolution of the immune system to provide both redundant and adaptable protection,^44,45^ suggesting that a similar pattern could also be employed by anti-Hib antibodies.

As concerns the DP2 binding mode, it is recognized by the CA4 hmAb in a fairly extended conformation, where the epitope region comprises its entire structure. Indeed, all of the sugar residues of the dimer are involved in polar and/or electrostatic interactions with the antibody, with the internal Ribf B′-Rib-ol A′′ groups giving the most important contributions. Since the analysis of the antibody binding pocket revealed the existence of potential space available to accommodate longer OS fragments, we also determined the 3D structure of the same Fab fragment complexed with the DP3 to evaluate whether longer OS may establish an increased number of interactions with the antibody and eventually engage interactions with the CA4 L chain. Surprisingly, the DP3 bound almost identically to DP2, only with the Fab H chain and involving only two RUs, suggesting that two RUs of the native Hib polysaccharide are required for optimal binding. This hypothesis is consistent with the STD-NMR results and with the weak electron density observed for the third RU of the DP3, especially for the ribose moiety, which protrudes outside the binding pocket and may explain the comparable affinity demonstrated by all the short OS fragments tested with this antibody.

The conformation of the Hib antigen in solution has been previously widely investigated, particularly through the application of NMR and molecular dynamics (MD) simulations. Indeed, earlier studies already highlighted the remarkable flexibility of the OS chains.^46−48^ As already mentioned, from the NMR analysis, it can be assessed that both DP2 and DP3 are fairly flexible molecules in solution with high conformational mobility along the torsional degrees of freedom at the linear ribitol chains, together with the pseudorotation at the five-membered ribose rings. However, once bound to the CA4 antibody, they adopt a well-defined conformation, in which the ribitol chain is perfectly accommodated into the antibody-binding pocket and the ribose ring displays a precise ring shape. Focusing on the ribose units, besides the detected anomeric mixture, the interconversion among ring conformations was further studied through J_H–H_ coupling analysis.^49^ For the β-furanose ribose, the ^3^T_2_ conformation is always more populated in solution for both the internal ribose residues and the reducing one, while for the α-furanose ribose, no preference for a specific ring shape was detected (Figure S7). Fittingly, the major ^3^T_2_ conformation is in full agreement with the X-ray-based bound conformation to the mAb. Furthermore, the ribitol moieties confer additional degrees of flexibility to the oligosaccharide and thus adaptability to the hmAb binding pocket. Altogether, these findings strengthen our hypothesis that two RUs represent an optimal saccharide length for antibody recognition.

Identification of the structural basis of carbohydrate minimal epitopes for antibody recognition represents a starting point for the future development of a modern vaccine with short specific oligosaccharides obtainable by synthetic, chemoenzymatic, or bioengineering methods. Despite several studies already reported for Hib CPS, the optimal saccharide length to ensure specific protection against infections remained unclear. Our results are in line with competitive ELISA experiments by Pillai et al. performed using differently sized Hib oligosaccharides as competitors for human monoclonal and polyclonal antibody binding that suggested that the DP2 oligomer may contain an optimal length for full filling of the antibody binding sites.^9^

Recently, it has been shown that four repeating units resemble the native polysaccharide in terms of immunogenicity and recognition by anti-Hib antibodies.^3^ Some previous immunogenic results on shorter oligosaccharide conjugates have reported that a length of three repeating units could be considered to be the optimal saccharide length to mimic the polysaccharide immunogenic epitope.^13,15^ In these studies, the synthetic conjugate dimer was able to elicit anti-PRP antibodies, although less than the respective trimer conjugates.^13^ The weakness of the immunogenicity trigged by the dimer could be explained by the low glycodensity on the carrier protein. A high degree of glycosylation could be fundamental for short oligosaccharides in order to allow multiple exposure of the epitope on the protein surface, leading to a robust antibody response.^18,19^ Thus, it is likely that two repeating units when multivalently exposed to a carrier protein are sufficient to behave as an effective glycoconjugate vaccine against Hib infections. Understanding the antigenic determinants of epitopes targeted by specific antibodies provides the basis for designing the next generation of a better and more effective Hib glycoconjugate vaccine by using technologies based on high-throughput chemical synthesis^50^ or innovative enzymatic approaches exploiting the increasingly detailed knowledge of CPS depolymerization processes.^51^

## Materials and Methods

### Selection of Oligosaccharides

- Oligosaccharides with a chain length of 2–5 repeating units were obtained by depolymerization of natural polysaccharide as published by Ravenscroft et al.^22^ Hib polysaccharide was hydrolyzed with acetic acid, and fragments of different lengths were separated by anion exchange chromatography using a Mono Q column.^22^ The chain length of each oligosaccharide was confirmed by ESI-MC while the point of cleavage between ribose and ribitol was confirmed by ^1^H/^31^P NMR: the absence of a phosphomonoester species detected by ^31^P NMR excludes cleavage at the level of phosphodiester linkage.
- A Hib polysaccharide with an average molecular weight of 28000 Da (avDP80) was obtained by treatment of the native polysaccharide with NaIO_4_ (1:0.08 mol/mol) for 30 min at 4 °C. After purification in size-exclusion G15 resin, sugar and aldehyde groups were measured by a colorimetric orcinol assay and microBCA based on a glucose standard curve, respectively. An activation of about 20% is measured from the ratio between moles of monomer and moles of aldehyde generated.

### HmAb and Fab Production

The recombinant human monoclonal antibody (hmAb) anti-Hib PS was produced by Takis SRL starting from published nucleotide sequences of a human IgG mAb.^20^ The hmAb, called CA4, was isolated from a fusion between a mouse–human heterohybridoma cell line and peripheral blood lymphocytes from an adult immunized with the Hib CPS vaccine. This hmAb demonstrated functional activity against Hib bacteria *in vitro* and *in vivo*.^20^ The Fab used comes from the hmAb mentioned above and was produced in two ways: hmAb digestion by papain enzyme (following the Pierce Fab Preparation Kit protocol) and production in mammalian cells (Expi293F). In detail, for the recombinant Fab production, heavy and light chain variable (V) region genes of hmAb of interest were amplified and then ligated by polymerase incomplete primer extension (PIPE) into mammalian expression plasmids (pcDNA 3.1, Thermofisher) containing the leader sequence for secretion and the constant region fragment of the IgG1 heavy or k-light chain, respectively. The plasmid carries in the coding sequence of the human IgG1 constant region of the heavy chain a truncation at the level of the AA responsible for pairing of the two long arms of antibodies, allowing only for the pairing of heavy and light chains, thus generating Fabs. These plasmids were then used to perform cotransfection and transient expression in Expi293F cells (Thermofisher) using Expifectamine (Thermofisher) according to the manufacturers’ instructions.

### Biotinylation of Oligosaccharides

Hib DP2, DP3, DP4, and avDP80 previously lyophilized were dissolved in a mixture of H_2_O and DMSO (1:9), subsequently biotin hydrazide (10 equiv compared to moles of oligosaccharides) and NaBH_3_CN (40 equiv compared to moles of oligosaccharides) were added to the oligosaccharide solution. The solution was kept at 37 °C for 3 days and purified through size exclusion in G10 resin. The product was checked by NMR and a colorimetric Q-Tag assay. Sugar quantification was measured by a colorimetric orcinol assay.

### MS Analysis

MS analysis was performed by a QtoF Premier mass spectrometer (Waters) with an ESI source and set to negative mode. The instrument was calibrated in the mass range of 113–1745 Da using sodium formate as a calibrant. The analysis was performed by direct infusion of sample diluted in 25% acetonitrile, and the spectrum was recorded with the following settings: polarity, ES-; analyzer, V mode; Np multiplier, 0.70; resolution, 8000; trigger threshold, 700; signal threshold, 35; capillary (kV), 1.5; sampling cone, 45.0; extraction cone, 4.0; ion guide, 2.5; source temperature (°C), 100; desolvation temperature (°C), 220; cone gas flow (L/h), 50.0; desolvation gas flow (L/h), 800.0; LM resolution, 4.7; HM resolution, 15.0; ion energy, 1.0; prefilter, 2.0; collision energy, 6.0; collision cell entrance, 2.0; collision cell exit, −12.0; collision gas flow, 0.45; scan time (s), 1.000; interscan time (s), 0.100; start mass 100.0 – end mass, 2500.0; data format, continuum; sensitivity, normal; and dynamic range, normal. Data acquisition and processing were performed by using Masslynx V4.2 software (Waters).

### SPR Analysis

Binding and kinetics were determined by SPR using a BIACORE X100 system. Biotinylated Hib OS avDP80, DP2, DP3, and DP4 were immobilized on a streptavidin-coated sensor chip (GE Healthcare) through a streptavidin–biotin capture using 1 M NaCl, 50 mM NaOH buffer for surface activation, and 1 M NaCl, 50 mM NaOH, and 50% isopropanol buffer to deactivate remaining active groups on the chip surface and remove noncovalently bound ligand. Biotinylated Hib avDP80 was used at 10 μg/mL, reaching an immobilized surface density of 277 resonance units. Binding competition was performed by incubating each competitor with the hmAb before injection. For each sample, the experiment was performed using a constant concentration of mAb and decreasing concentrations (2-fold dilutions) of competitor. The ability of the competitor to inhibit the mAb binding to immobilized Hib avDP80 is expressed as a percentage or reduction of the binding level compared to not-competitive mAb.

Biotinylated Hib DP2, DP3, and DP4 were used at 100 nM, reaching immobilized surface densities of 52, 18, and 29 resonance units, respectively. Kinetics experiments with the Fab fragment were performed using 2× diluted solutions. All experiments were conducted in 10 mM HEPES (pH 7.2), 150 mM NaCl, 3 mM EDTA, and 0.005% Tween20 at 25 °C and at a flow rate of 45 μL/min. After each cycle of hmAb and Fab flow, the chips were regenerated with 3.5 M MgCl_2_ and a contact time of 120 s. Sensorgram data were analyzed by using BIAevaluation software (Biacore).

### NMR Studies

^31^P NMR experiments were acquired using a Bruker AVANCE 2 400 MHz spectrometer (Bruker Inc.; Billerica, MA, U.S.A.). ^1^H–^13^C and ^1^H NMR experiments were acquired using a Bruker AVANCE 2 800 MHz spectrometer (Bruker Inc.; Billerica, MA, U.S.A.) equipped with a cryo-probe, both for ligand characterization and ligand–protein interaction studies. All of the NMR samples were prepared in either D_2_O/H_2_O or pure D_2_O solution, and samples were transferred to 5 mm Shigemi NMR tubes (New Era Enterprises, Vineland, NJ, U.S.A.). Data acquisition and processing were performed using TOPSPIN 3.5 software.

The hmAb was purified through PD-10 desalting columns packed with Sephadex G-25 resin and exchanged in 50 mM Tris-HCl buffer pH 8.0 through a 2 mL Zeba Spin desalting column. For the NMR experiment, the buffer of CA4 hmAb was exchanged with deuterated phosphate saline buffer (50 mM sodium phosphate, 150 mM NaCl, pD 8.0) using a Vivaspin centrifugal concentrator with a cutoff of 100 kDa. The final pD was measured with a Crison Basic 20 pH meter (Crison Instruments SA, Barcelona, Spain) and adjusted by adding the required amounts of NaOD and DCl. The stability of the antibody after buffer exchange was checked by ^1^H NMR experiments, and the protein profile showed good chemical shift dispersion, indicating that the tertiary structure was intact.

For the STD-NMR experiments, the antibody was employed at a final concentration of 10 μM and the ligand:antibody ratio was set at 50:1. The temperature was set at 298 K during the acquisition. The STD sequence was selected from an in-house library and included a T1rho filter. The off-resonance frequency was set at 100 ppm, and the on-resonance frequency was set at δ 0.6 ppm. The experiments were acquired with 2 s of saturation time, 3 s of relaxation delay, and 2880 scans. The final STD spectra were obtained by subtracting the on-resonance spectrum from the off-resonance spectrum. The STD Amplification Factor (STD-AF) was calculated based on the comparison between the signals of the STD spectrum and those of the off-resonance spectrum. The STD% was calculated by normalization of the whole set of STD factors against the highest value for each ligand.

### X-ray Crystallography

#### Protein Crystallization

Human Fab CA4 was purified in 20 mM Tris-HCl and 150 mM NaCl, pH 8.0, and concentrated to 20 mg/mL using centrifugal filter devices with a 10 kDa cutoff Amicon membrane. DP2- and DP3-Fab complexes (15:1 saccharide/Fab molar ratio) were prepared by incubating the lyophilized sugars with the Fab solution at room temperature. Crystallization screenings were performed using a sitting-drop vapor-diffusion format at 293 K by mixing equal volumes (200 nL) of the complexes with crystallization reservoir solutions using a Crystal Gryphon liquid handling robot (Art Robbins Instruments). DP2-Fab crystals were obtained after 6 days using a reservoir made of 0.01 M zinc sulfate heptahydrate, 0.1 M MES 6.5, and 25% v/v PEG 500 MME. DP3-Fab crystals were obtained after 4 days in a reservoir of 0.01 M nickel(II) chloride hexahydrate, 0.1 M Tris 8.5, and 20% w/v PEG 2000 MME. All crystals were soaked in a cryoprotection solution composed of 25% v/v ethylene glycol and 75% reservoir solutions and flash-cooled in liquid nitrogen for subsequent data collection at 100 K.

#### Structure Determination

X-ray diffraction data of the Fab-DP2 complex were collected at Diamond Light Source (Didcot, Oxforshire, U.K.) on beamline I03 equipped with an Eiger2 XE 16 M detector. For the Fab-DP3 complex, diffraction data were collected at the European Synchrotron Radiation Facility (ESRF), Grenoble, France, on beamline ID30A-1 using a PILATUS3 2 M detector. Diffraction data were integrated with DIALS^52^ and scaled with Aimless^53^ software from the CCP4 program suite.^54^ Crystals of both complexes belonged to the orthorhombic C2221 space group, with approximate cell parameters of *a* = 60.67 Å, *b* = 131.59 Å, *c* = 145.1 Å, and one copy of the Fab-OS complex in the asymmetric unit. The structure of the Fab-DP2 complex was determined by molecular replacement in Phaser,^55^ using the coordinates of Fabs with PDB codes 3KYM and 6AZM as template models. The refined coordinates of Fab CA4 were used for molecular replacement of the DP3 complex. Refinement of both structures and manual model building were performed using Phenix.refine^56^ and Coot,^57^ respectively.

#### Structure Quality

The final models were inspected and validated with Molprobity.^58^ The buried surface areas and the root-mean-square displacements were calculated with PISA^59^ and Coot,^57^ respectively. Atomic interactions and contacts between the oligosaccharides and the Fab were calculated with MOE software (version 2020.0901, Chemical Computing Group, Montreal, QC, Canada) and manually inspected. Figures were generated using PyMOL (PyMOL Molecular Graphics System, version 2.5.2; Schrödinger, LLC; http://www.pymol.org) and CCP4 mg.^60^ Data collection and refinement statistics are listed in Table S1.

## Supporting Information Available

Supporting NMR, STD-NMR, and ESI-MS data; MD simulation studies; chemical representation of Hib oligosaccharide and scheme of sugars formed in water and biotinylation; and supporting X-ray data (PDF)

## Accession Codes

The final coordinates and structure factors of Fab CA4 complexed with DP2 and DP3 OS have been deposited in the Protein Data Bank with accession codes 8RDA and 8RDF, respectively.
