# Supplementary material for: A Multidisciplinary Structural Approach to the Identification of the Haemophilus influenzae Type b Capsular Polysaccharide Protective Epitope
Source: ACS Cent Sci. 2024 Feb 22;10(5):978–87. doi: 10.1021/acscentsci.3c01515 (PMC11117310; doi:10.1021/acscentsci.3c01515)
Supplement: Supplementary file 1 — oc3c01515_si_001.pdf [file oc3c01515_si_001.pdf]

SUPPORTING INFORMATION (SI)

A multidisciplinary structural approach for the identification of *Haemophilus influenzae* type b capsular polysaccharide protective epitope

Francesca Nonne[a], Lucia Dello Iacono[b], Sara Bertuzzi[c], Luca Unione[c,d], Daniela Proietti[b], Nathalie Norais[b], Immaculada Margarit[b], Roberto Adamo[b], Jesús Jiménez-Barbero[c,d,e,f], Filippo Carboni[b], Maria Rosaria Romano[b*]

Affiliation

[a] GSK Vaccines Institute for Global Health, Siena, Italy

[b] GSK, Siena, Italy

[c] CIC bioGUNE, Basque Research Technology Alliance, BRTA, Bizkaia Technology Park, 48160 Derio, Spain

[d] IKERBASQUE, Basque Foundation for Science and Technology, Euskadi Plaza 5, 48009 Bilbao, Spain

[e] Department of Organic & Inorganic Chemistry, Faculty of Science and Technology, University of the Basque Country, 48940 Leioa, Spain

[f] Centro de Investigación Biomédica En Red de Enfermedades Respiratorias, Madrid, Spain

*Corresponding Author email: maria.r.romano@gsk.com

A.

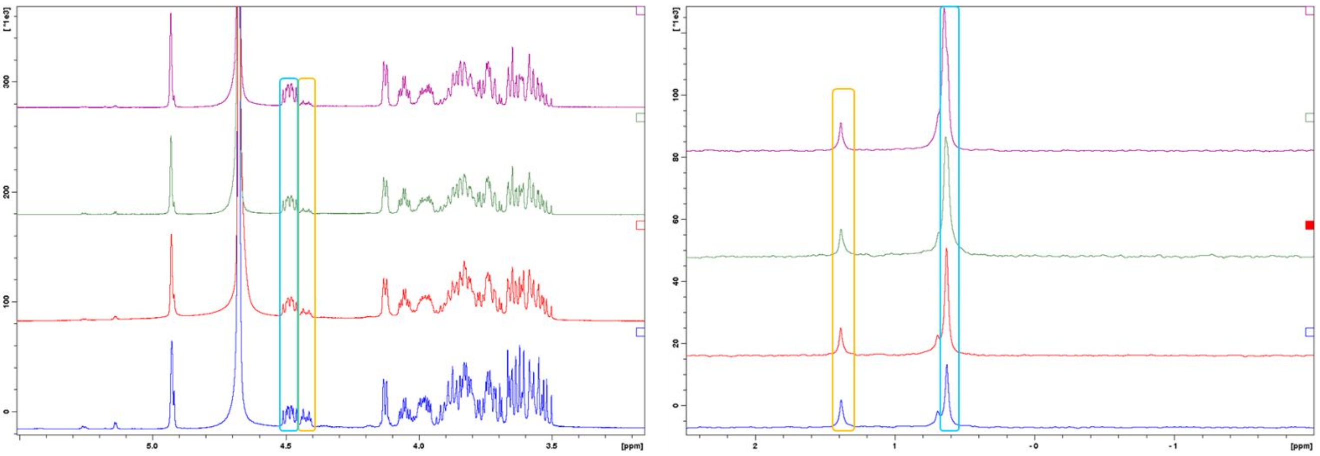

B.

OS	Formula	Exact Mass	Calculated $[M-H]^{-1}$	Detected $[M-H]^{-1}$	Calculated $[M-2H]^{-2}$	Detected $[M-2H]^{-2}$	Calculated $[M-3H]^{-3}$	Detected $[M-3H]^{-3}$	Calculated $[M-4H]^{-4}$	Detected $[M-4H]^{-4}$
DP2	$C_{20}H_{40}O_{23}P_2$	710.14	709.14	709.13	354.06	354.06	235.71	n.d.	176.53	n.d.
DP3	$C_{30}H_{59}O_{34}P_3$	1056.21	1055.20	1055.20	527.10	527.10	351.06	351.06	263.04	n.d.
DP4	$C_{40}H_{78}O_{45}P_4$	1402.28	1401.27	1401.25*	700.13	700.13	466.42	466.42	349.56	349.53
DP5	$C_{50}H_{97}O_{56}P_5$	1748.34	1747.34	1747.34*	873.16	873.16	581.77	581.78	436.08	436.07

n.d.=not detected

*obtained from the deconvoluted spectrum

Figure S1. A) ^{1}H-NMR spectra (left) and ^{31}P-NMR spectra (right) of the produced Hib oligosaccharide fragments: DP2 (blue), DP3 (red), DP4 (green), DP5 (purple). Signals of H3-ribose within the chain and at the reducing terminal units are indicated in light blue and yellow boxes, respectively (left panel). The relative intensity of the reducing terminal H3-ribose signal decreased with increasing chain length. Signals of phosphate groups of terminal RU and within the chain are circled yellow and light blue, respectively (right panel). **B)** DP2-DP5 mass detected by negative mode ESI-MS.

A.

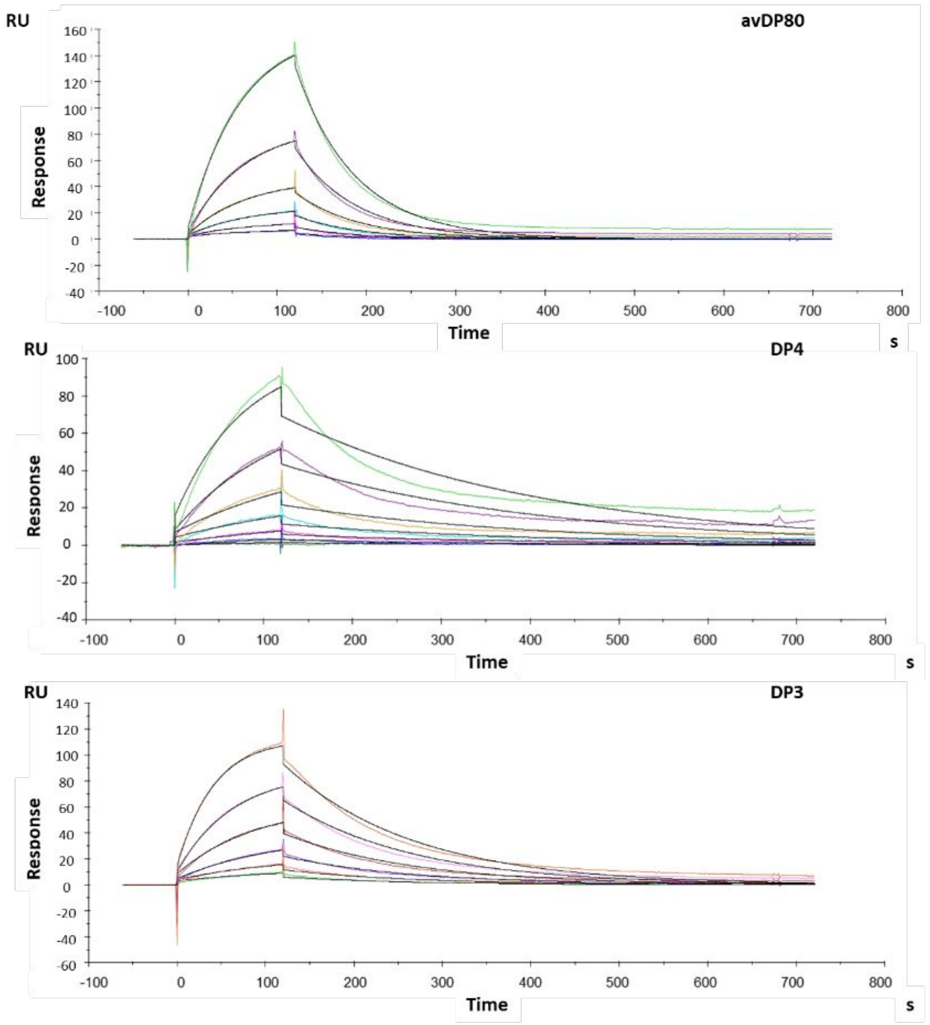

B.

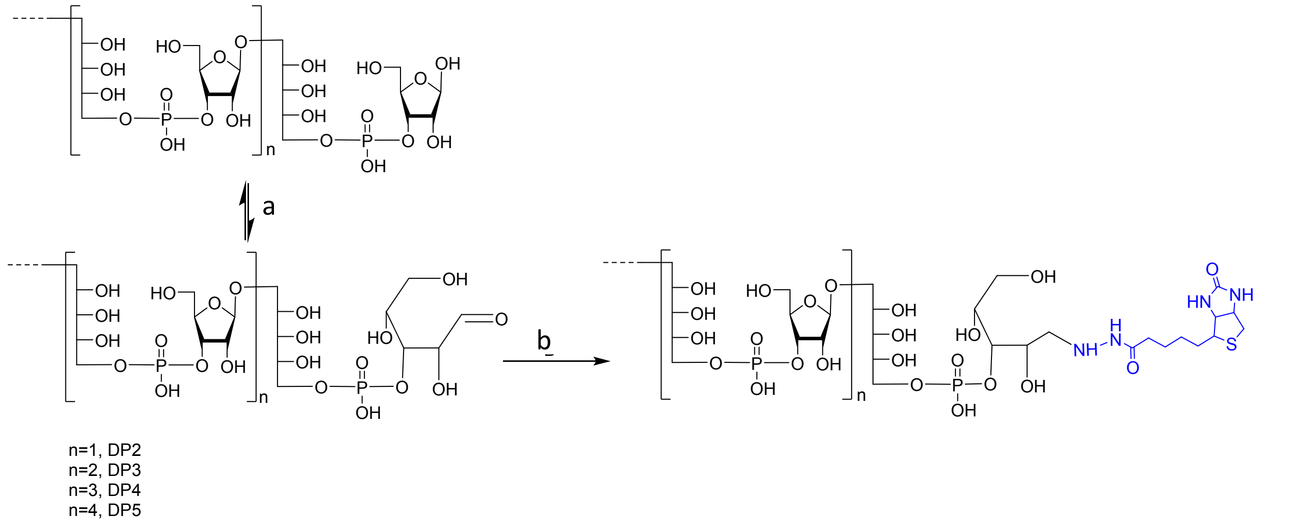

Figure S2. A) Binding kinetics of Fab CA4 to avDP80, DP4 and DP3 fragments by SPR. **B)** Sugar forms in water [25] (a) and biotinylation (b)

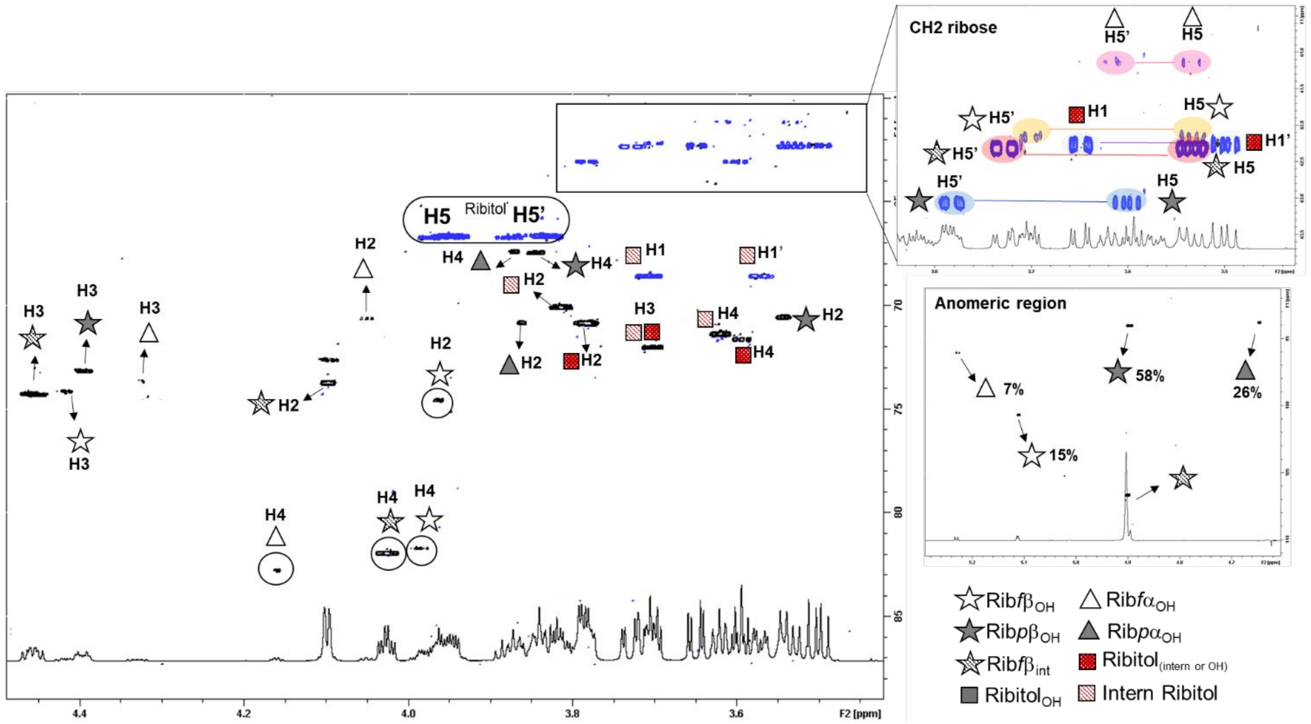

Figure S3. ^{1}H-^{13}C full HSQC spectrum of Hib DP3 1 mM in D_2O with the relative peak assignment. The experiment was acquired with 500 µL of sample transferred into a 5 mm NMR tube. The assignment for Hib DP2 is identical to the one here reported for DP3.

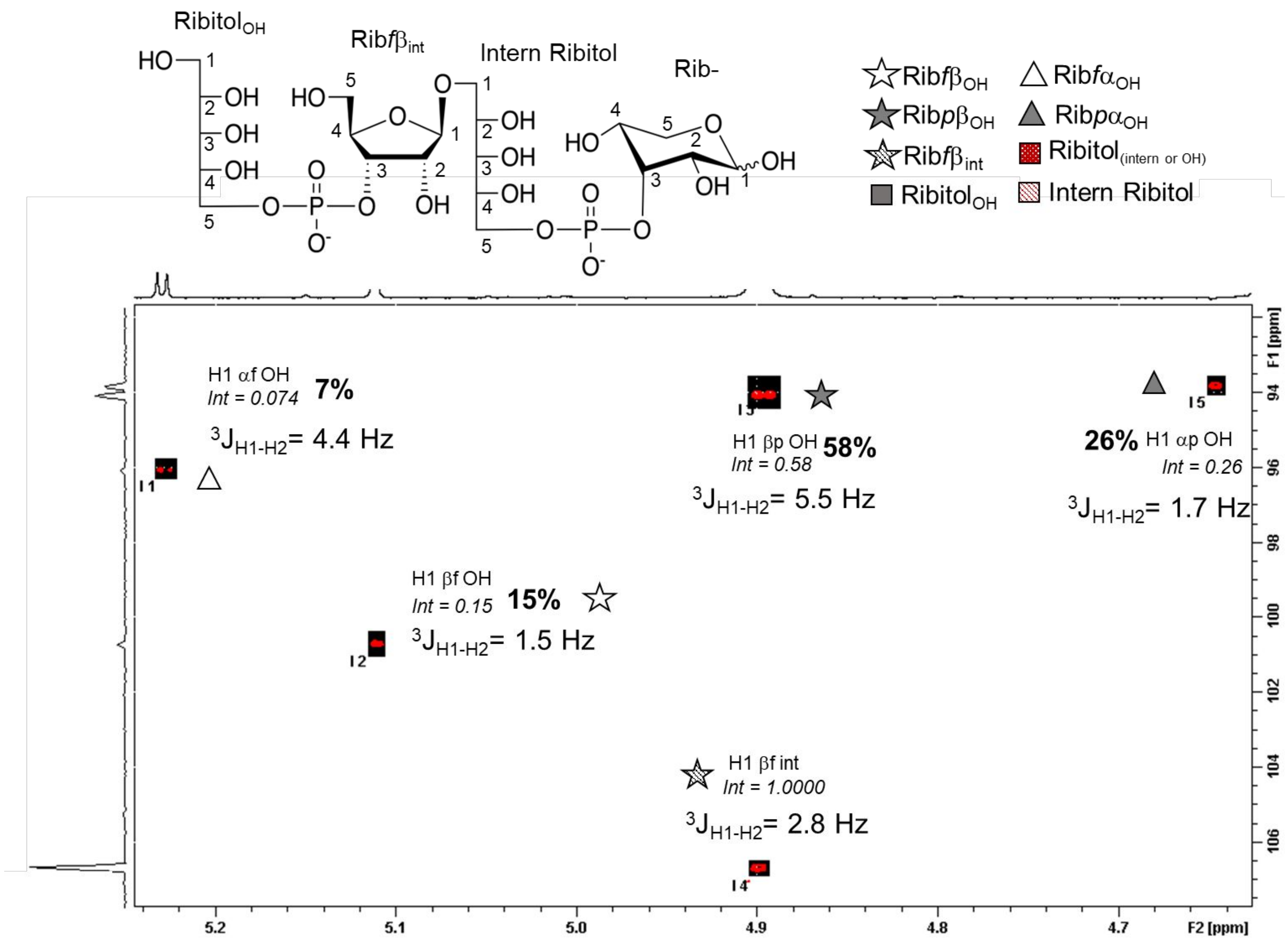

Figure S4. Zoom of the ^1H-^{13}C HSQC spectrum of Hib DP3 1 mM in D_2O on the anomeric region. The peak assignment is reported, together with the calculated $^3J_{H1-H2}$ couplings, the integrals for each signal and the relative percentage for each anomer. The experiment was acquired with 500 µL of sample transferred into a 5 mm NMR tube.

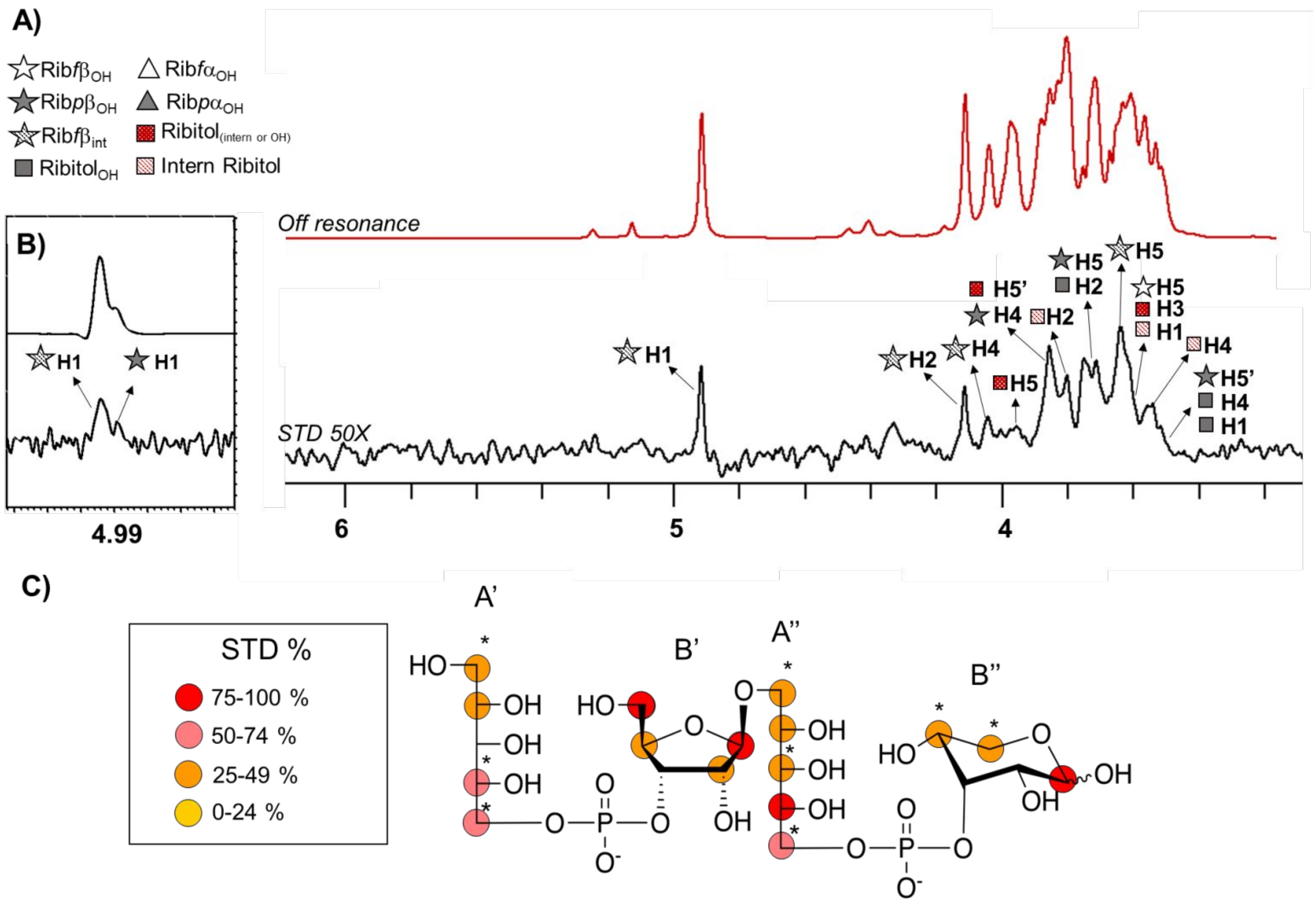

Figure S5. A) STD NMR experiments performed for the complex Hib DP2-CA4 hmAb. Off-resonance spectrum (irradiation at δ 100 ppm, top panel) and STD spectra obtained from aliphatic irradiation (δ 0.6 ppm) and processed applying exponential line-broadening. The main ^{1}H NMR signals are annotated in the STD spectrum and the pictorial notation for each residue is reported. The antibody/ligand molar ratio was 1:50, with the CA4 hmAb being at a concentration of 10 μM. The STD NMR experiments were acquired with 2 s of saturation time, 3 s of relaxation delay, and 2880 scans at 298 K. **B**) Expansion of the STD in the anomeric region of protons H1 Ribfβ_{int} and Rib*p*β_{OH} processed omitting the exponential multiplication for resolution enhancement. **C**) Representation of the epitope map disclosed by the analysis of the STD-NMR experiments of Hib DP2-CA4 hmAb and Hib DP3-CA4 hmAb complexes. The color legend of associated to STD% values is also reported.

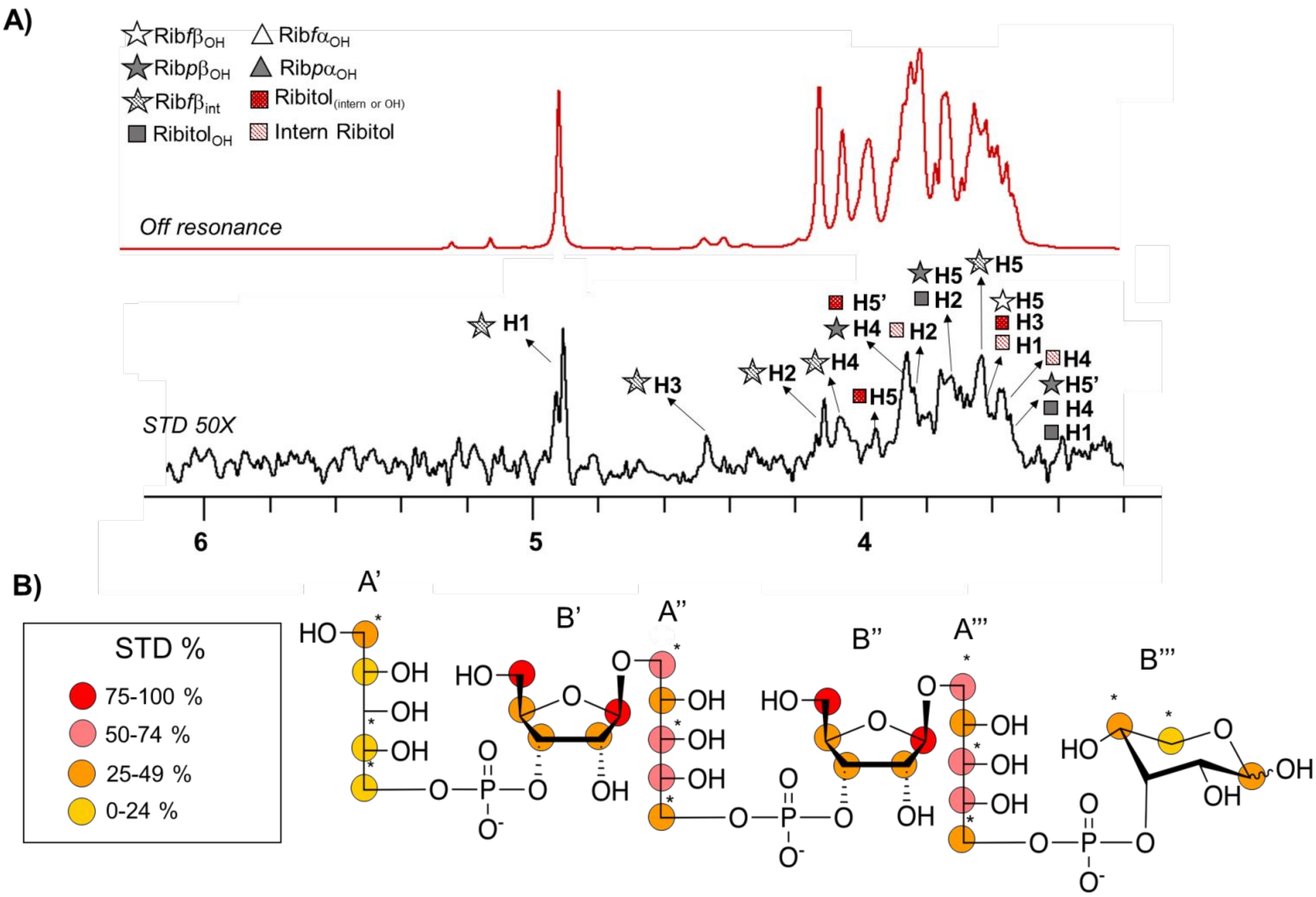

Figure S6. STD NMR experiments performed for the complex Hib DP3-CA4 hmAb. Off-resonance spectrum (irradiation at δ 100 ppm, top panel) and STD spectra obtained from aliphatic irradiation (δ 0.6 ppm) and processed applying exponential line-broadening. The main ^{1}H NMR signals are annotated in the STD spectrum and the pictorial notation for each residue is reported. The antibody/ligand molar ratio was 1:50, with the CA4 hmAb being at a concentration of 10 μM. The STD NMR experiments were acquired with 2 s of saturation time, 3 s of relaxation delay, and 2880 scans at 298 K. **B**) Representation of the epitope map disclosed by the analysis of the STD-NMR experiments of Hib DP3-CA4 hmAb and Hib DP3-CA4 hmAb complexes. The color legend associated to the STD% values is also reported.

A)

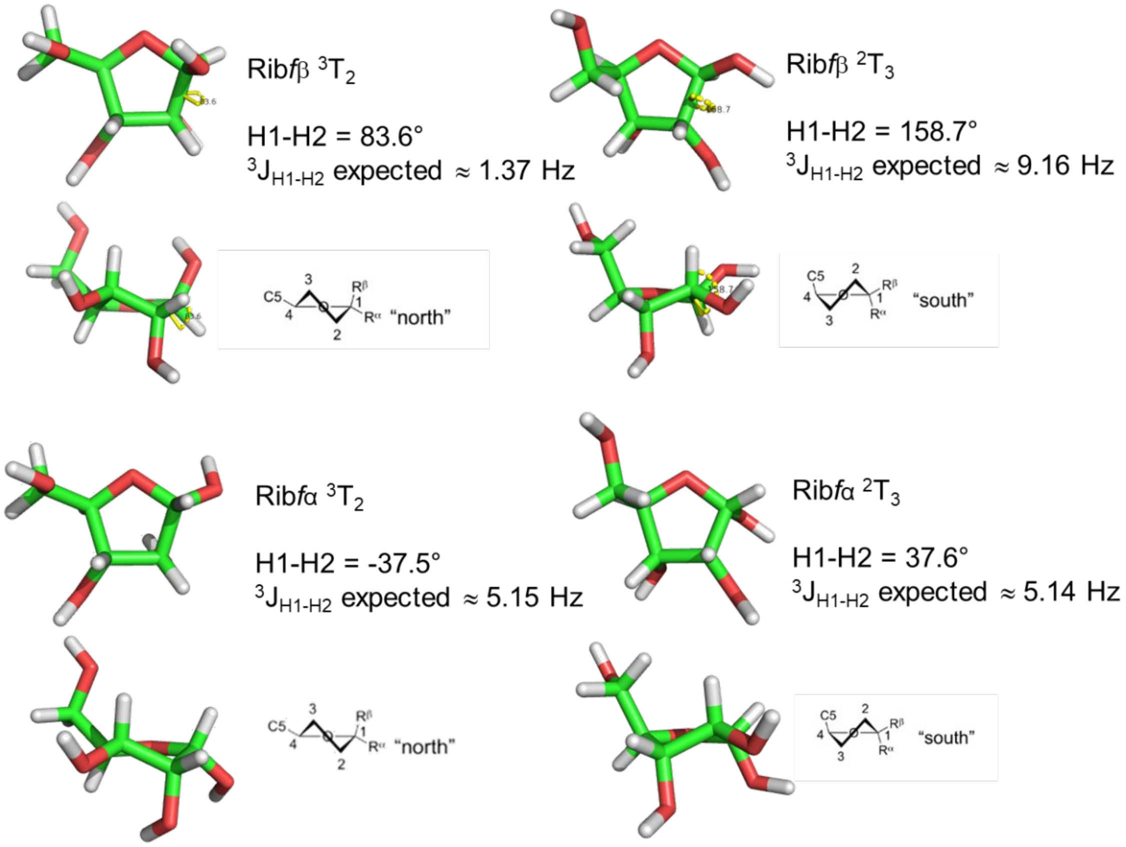

B)

	**Experimental** $^3J_{H1-H2}$	$p_{Rf\beta}$ **3T2**	$p_{Rf\beta}$ **2T3**
Internal Rib*f*β	2.8 Hz	0.82	0.18
Reducing Rib*f*β	1.5 Hz	0.98	0.02

	**Experimental** $^3J_{H1-H2}$	$p_{Rf\beta}$ **3T2**	$p_{Rf\beta}$ **2T3**
Reducing Rib*f*α	4.4 Hz	0.5	0.5

Figure S7. A) Study of the interconversion of ring conformations performed through J coupling analysis. Representation of the possible $^3T_2$ and $^2T_3$ conformations for Rib*f*β (on the left) and Rib*f*α (on the right) with the $^3J_{H1-H2}$ expected values reported. **B**) Table reporting the measured experimental $^3J_{H1-H2}$ and the relative population for each moiety.

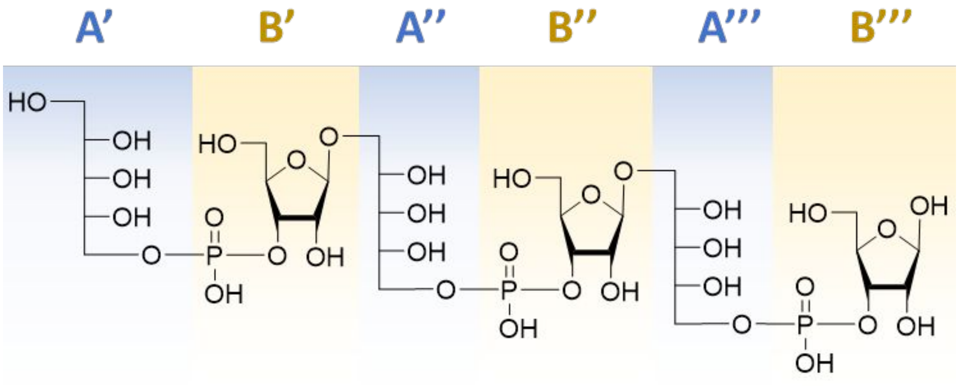

Figure S8. Ribitol and Ribose moieties of the DP3 fragment are individually indicated as A’, B’, A’’, B’’, A’’’, B’’’ starting from the Ribitol of the first repeating unit.

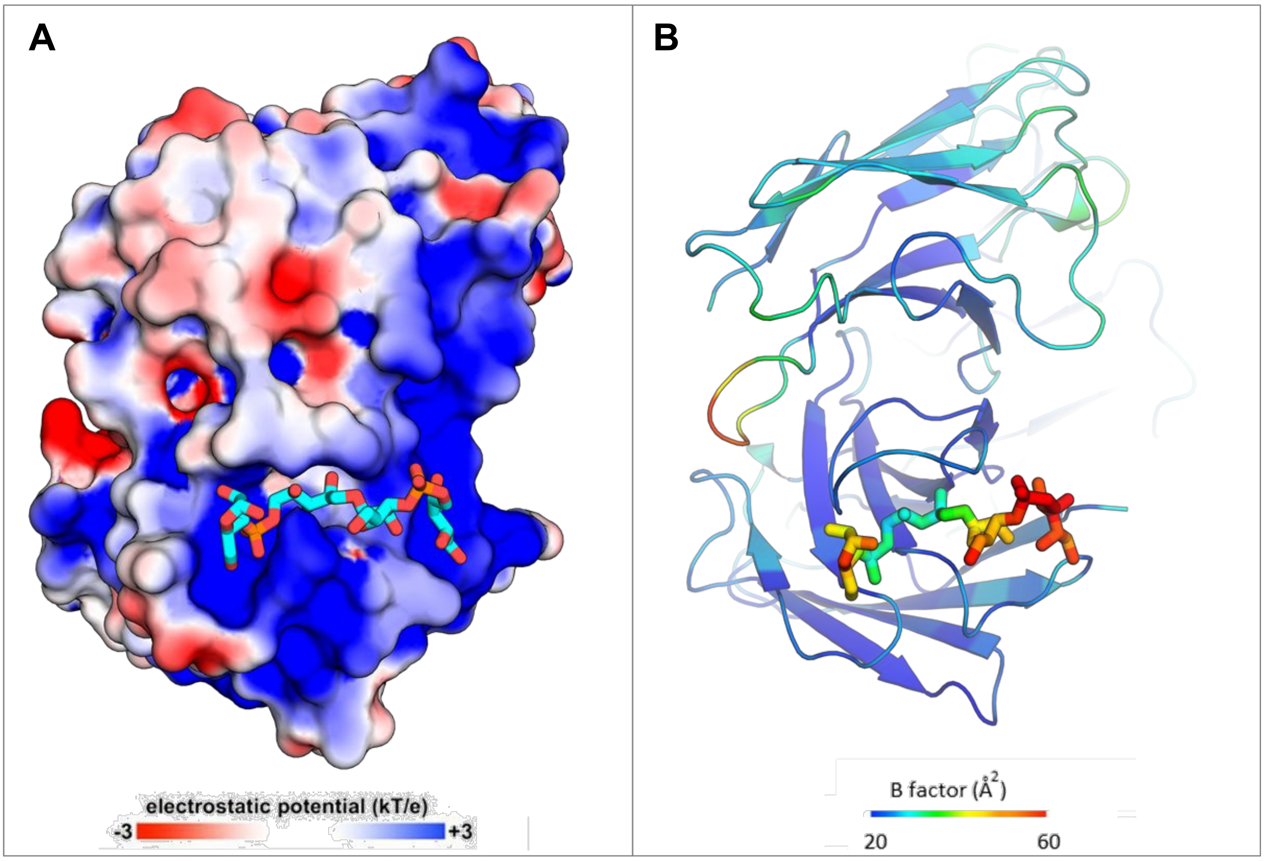

Figure S9. A) Electrostatic surface representation of the Fab CA4 (ranging from −3 kT/e [red] to +3 kT/e [blue]), calculated by APBS (adaptive Poisson-Boltzmann solver) methods [59] using Pymol. **B)** B-Factor values for Fab and Hib DP2. Crystallographic B factors represent temperature-dependent vibrations from average atomic positions. The color bar ranges from low B factor (blue, less flexible) to high B factor (red, more flexible).

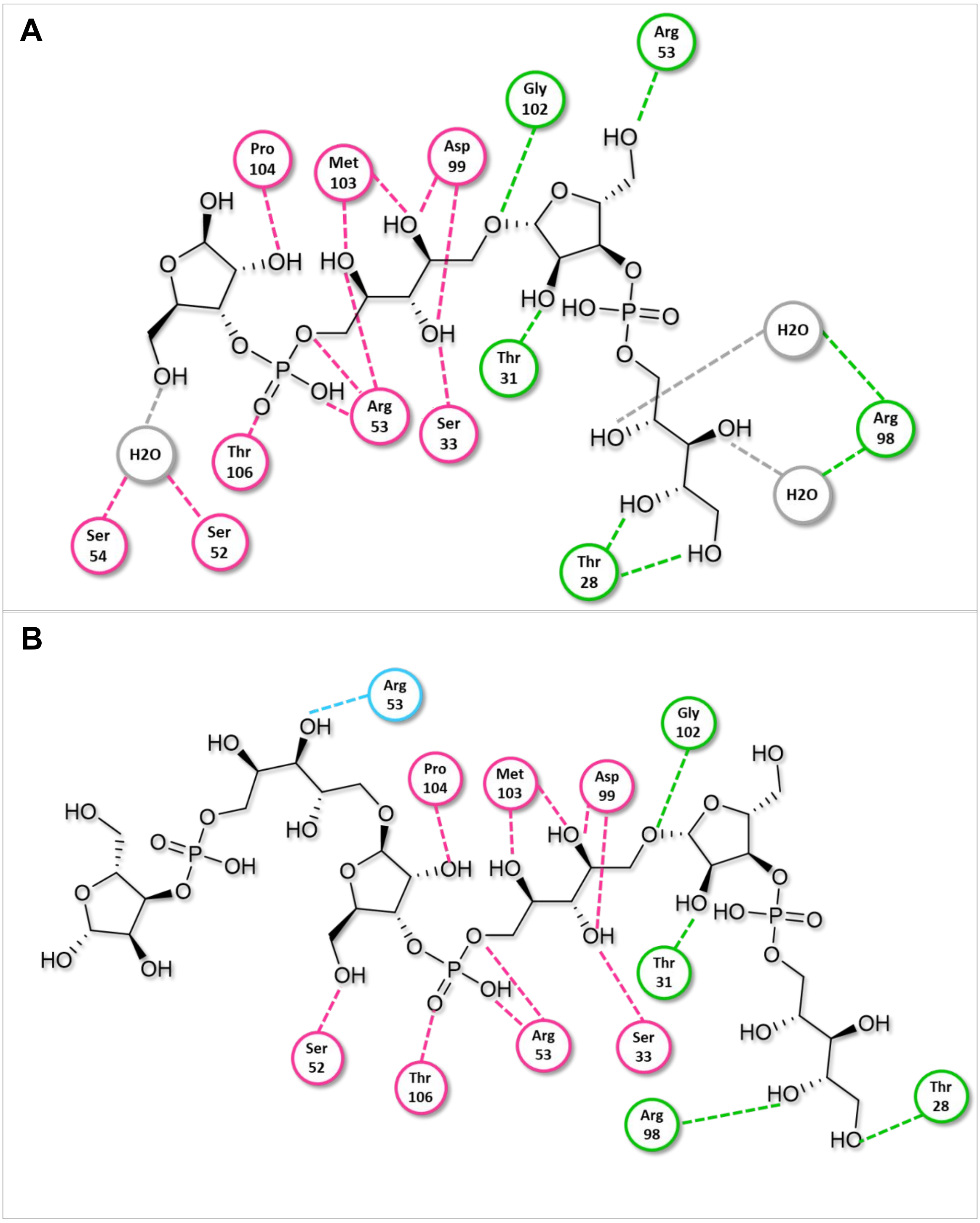

Figure S10. Schematic representation of DP2 **(A)** and DP3 **(B)** interactions. Hydrogen bonds are indicated by dotted lines. Interactions of the OS fragments with Fab residues are shown in green (Rib-ol A'-Ribf B'), pink (Rib-ol A''-Ribf B'') and light blue (Rib-ol A'''-Ribf B'''). Water-mediated interactions are represented in grey.

Table S1. Data collection and refinement statistics

	**Fab CA4-DP2** (PDB id 8RDA)	**Fab CA4-DP3** (PDB id 8RDF)
**Crystal**		
Space group	C 2 2 2_1	C 2 2 2_1
Cell dimensions *a, b, c* (Å)	60.66, 131.59, 145.1	60.80, 131.22, 144.95
**Data collection**[a]		
Beamline	DLS I03	ESRF ID30A-1
Wavelength (Å)	0.976	0.965
Resolution (Å)	51.50-2.29 (2.33-2.29)	65.64- 2.74 (2.87-2.74)
Total reflections	207267 (5801)	53118 (6956)
Unique reflections	26582 (1252)	15060 (2007)
R_{merge}	0.16 (1.16)	0.18 (0.94)
R_{meas}	0.19 (1.45)	0.21 (1.11)
$I/\sigma(I)$	7.3 (1.1)	5 (1.2)
$CC_{1/2}$	0.99 (0.38)	0.94 (0.58)
Completeness (%)	99.7 (93.4)	96.8 (98.1)
Redundancy	7.8 (4.6)	3.5 (3.5)
Wilson B-factor (Å)	37.09	36.55
**Refinement**		
Resolution (Å)	51.50-2.29	65.64-2.74
No. reflections	26531	14988
R_{work} / R_{free}	22.6/27	22.6/27.7
No. atoms		
Protein	3307	3282
Ligands	50	68
Water	114	54
B factors		
Protein (Heavy/ Light chains)	43.78/ 47.96	40.88/46.20
Ligand (carbohydrate)	65.57	52.44
Water	44.17	37.40
R.m.s. deviations		
Bond lengths (Å)	0.002	0.003
Bond angles (°)	0.62	0.69
Clash scores	3.2	7.2
Ramachandran[#]		
Favored (%)	96.53	95.08
Allowed (%)	3.24	3.98

[a] Values in parentheses are for highest-resolution shell
[#] Measured using Molprobity
